# Supplementary material for: Diet-omics in the Study of Urban and Rural Crohn disease Evolution (SOURCE) cohort
Source: Nat Commun. 2024 May 4;15:3764. doi: 10.1038/s41467-024-48106-6 (PMC11069498; doi:10.1038/s41467-024-48106-6)
Supplement: Supplementary file 1 — Supplementary Information [file 41467_2024_48106_MOESM1_ESM.pdf]

Diet-omics in the Study of Urban and Rural Crohn disease Evolution (SOURCE) cohort

Tzipi Braun[1&], Rui Feng[2,3&], Amnon Amir[1], Nina Levhar[1], Hila Shacham[1], Ren Mao[2], Rotem Hadar[1], Itamar Toren[1,4], Yadid Algavi[5], Kathleen Abu-Saad[6], Shuoyu Zhuo[2], Gilat Efroni[1], Alona Malik[1], Orit Picard[1], Miri Yavzori[1], Bella Agranovich[7], Ta-Chiang Liu[8], Thaddeus S Stappenbeck[9], Lee Denson[10], Ofra Kalter-Leibovici[4,6], Eyal Gottlieb[11], Elhanan Borenstein[4,12,13], Eran Elinav[14,15], Minhu Chen[2#], Shomron Ben-Horin[1,5#], Yael Haberman[1,5,8#*]

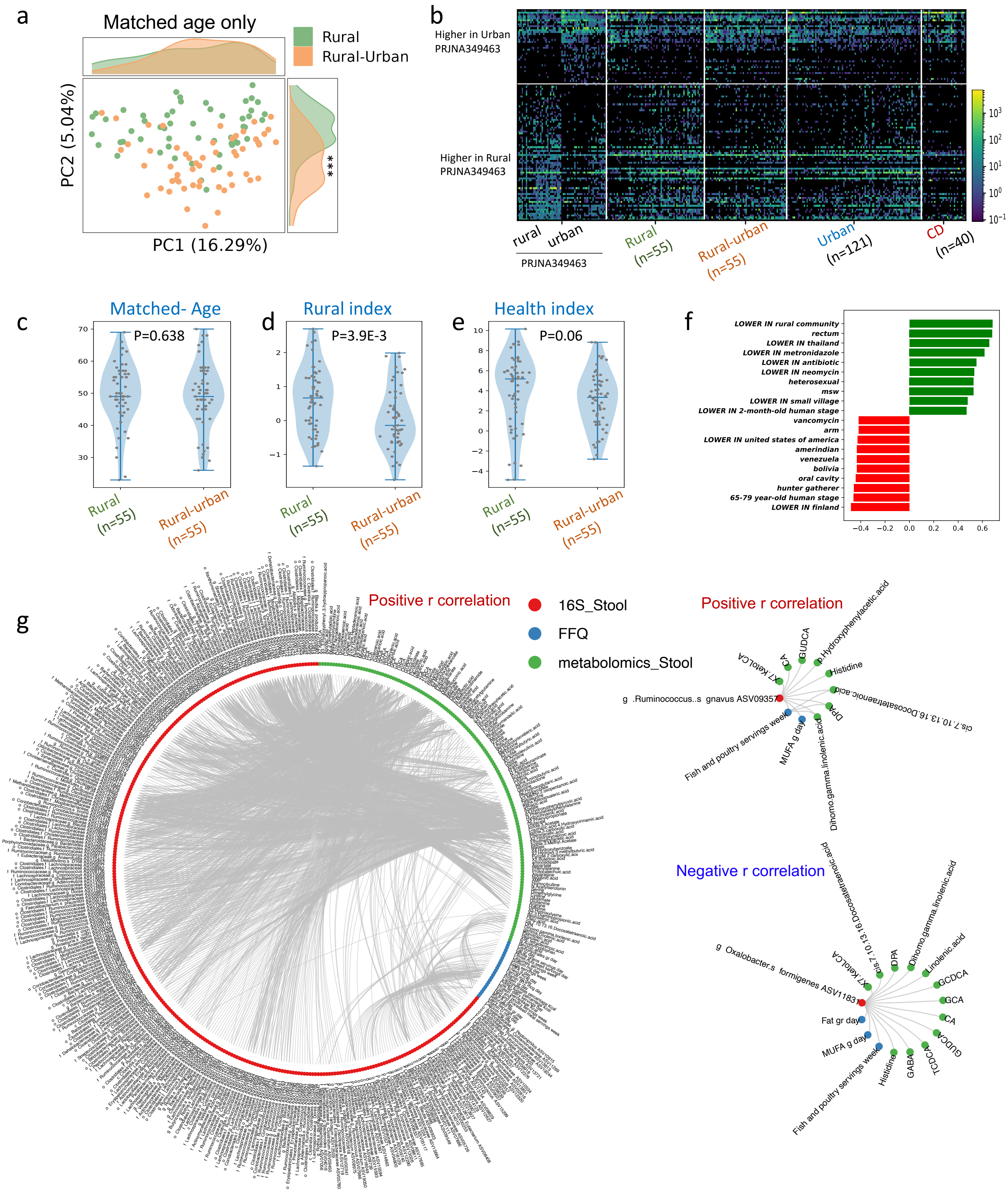

Supplementary Fig. 1. Microbial population in rural residents who spend more than half of their time in an urban environment while controlling for age. Age was significantly higher in rural samples (Mann-Whitney p. value = 9.38e-8), we therefore created an age-matched sub-cohort with 55 rural and 55 rural-urban samples, to confirm that our findings were not the result of an age bias. **a.** Unweighted UniFrac PCoA plot of 110 age-matched rural and rural-urban China 16S stool samples, colored by group. Histograms show the distribution of samples and group on PC1 and PC2.

b. A heatmap showing ASVs with significant differential abundance between rural and urban samples from BioProject PRJNA349463 and their expression in our China 16S samples. Each row represents a different ASV and each column is a different sample. Those taxa were used to generate a 'rural index' that was applied to our cohort. **(c-e)** Violin plots showing age **(c)**, rural index **(d)**, and health index **(e)**, between age-matched rural (n=55) and rural-urban (n=55) samples, with Mann-Whitney p. values of 0.638, 3.9E-3, and 0.06 respectively. **(f)** dbBact enrichment terms (based on other experiments) of ASVs higher or lower in rural vs rural-urban from **Fig. 2g. g.** Circus plot of HAllA positive correlations within all rural (n=162) between FFQ components, metabolites, and 16S stool bacterial ASVs (FDR ≤ 0.25). For 16S and diet (n=162, 255 total significant associations between, FDR≤0.25For metabolomics vs FFQ (n=40, 23 significant associations), and for microbiome and the fecal metabolome (n=40, 2675 significant associations). Positive correlation of all metabolites and FFQ components significantly correlated with the bacteria *R. gnavus* (right up), and negative correlation of all metabolites and FFQ components significantly anti-correlated with the bacteria *Oxalobacter formigenes* as indicated. *p< 0.05, **p<0.01, ***p<0.001, Mann-Whitney test.

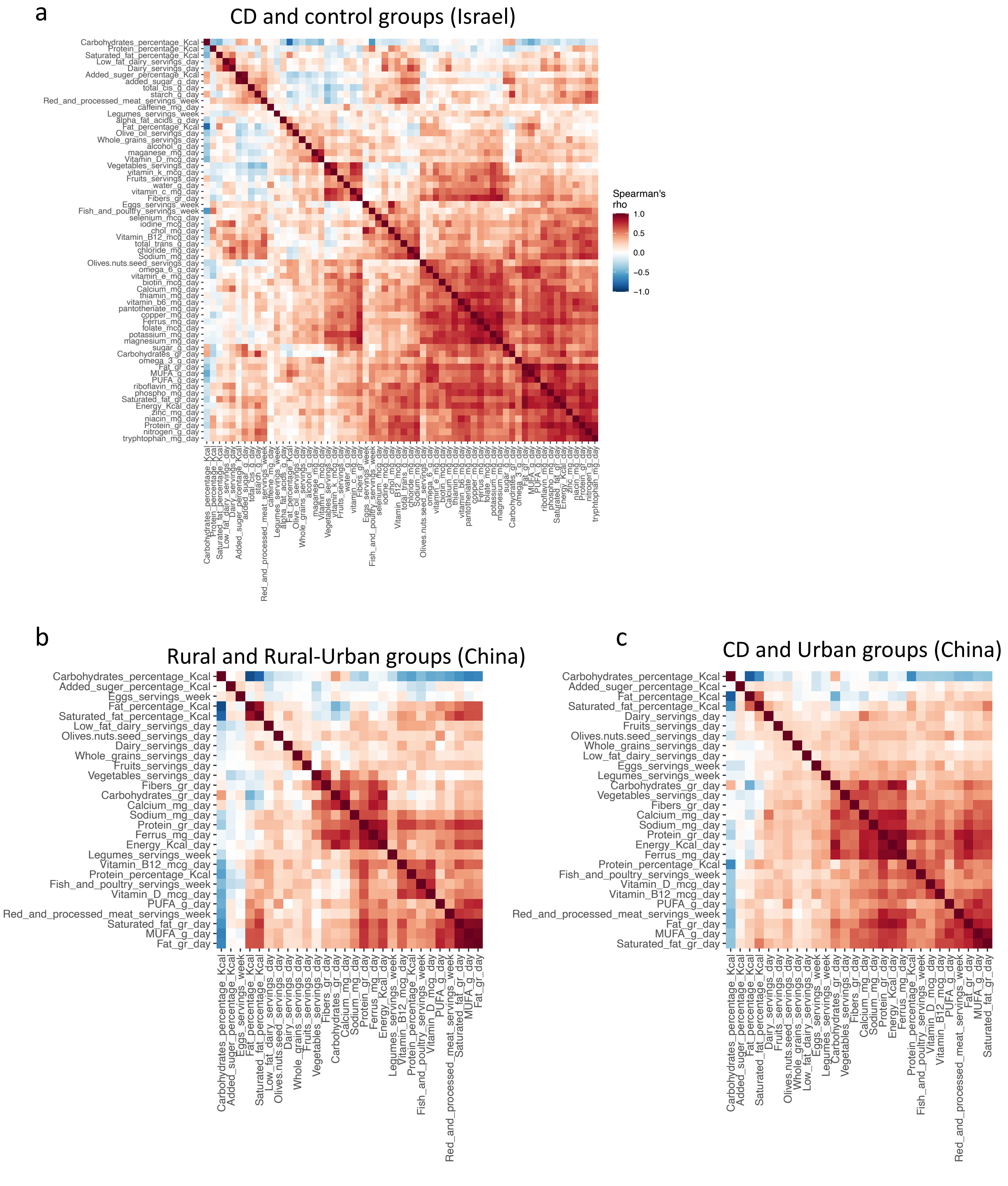

Supplementary Fig. 2. Correlations between FFQ dietary components. Heatmaps showing Spearman's correlation rho values for the correlation between different FFQ dietary components, in **(a)** Israel samples (samples n=47, FFQ features n=62), **(b)** China rural and rural-urban samples (samples n=148, FFQ features n=28), and **(c)** China urban and CD samples (samples n=160, FFQ features n=28).

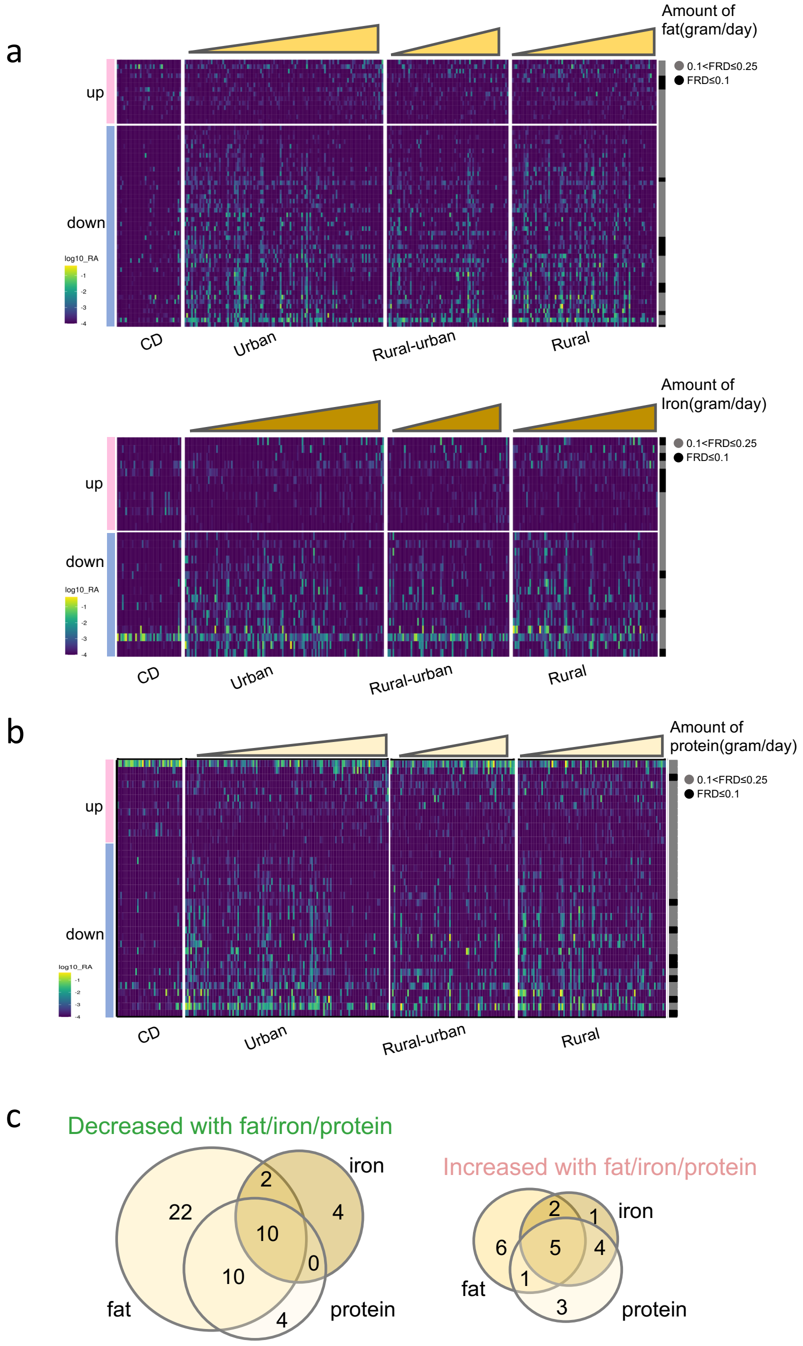

Supplementary Fig. 3. Protein, iron, and fat are linked with microbial variations in China. a. A heatmap showing ASVs that were significantly associated ($p<0.05$, FDR $\leq$ 0.25, black mark on the right indicates those with FDR$\leq$0.1) with total fat (up) and iron (down) consumption, using a maaslin2 analysis controlling for age, gender, and group in China (rural, rural-urban, and urban). This analysis was performed within only the 283 control samples from China, with the CD subjects (n=40) shown for comparison. Each row represents a different ASV, ordered by the effect size, and each column a different sample, ordered by fat (up) or iron (down) consumption within the indicated group **b.** As in **a** but showing ASVs that were significantly associated with total protein consumption. **c.** Venn plots showing the overlap between the taxa decreased in fat, iron, and protein (left) adding 4 ASVs to the 48 in **Fig. 3**, and the taxa increased with protein, fat, and iron consumption (right) adding 3ASVs to the 19 in **Fig. 3.**

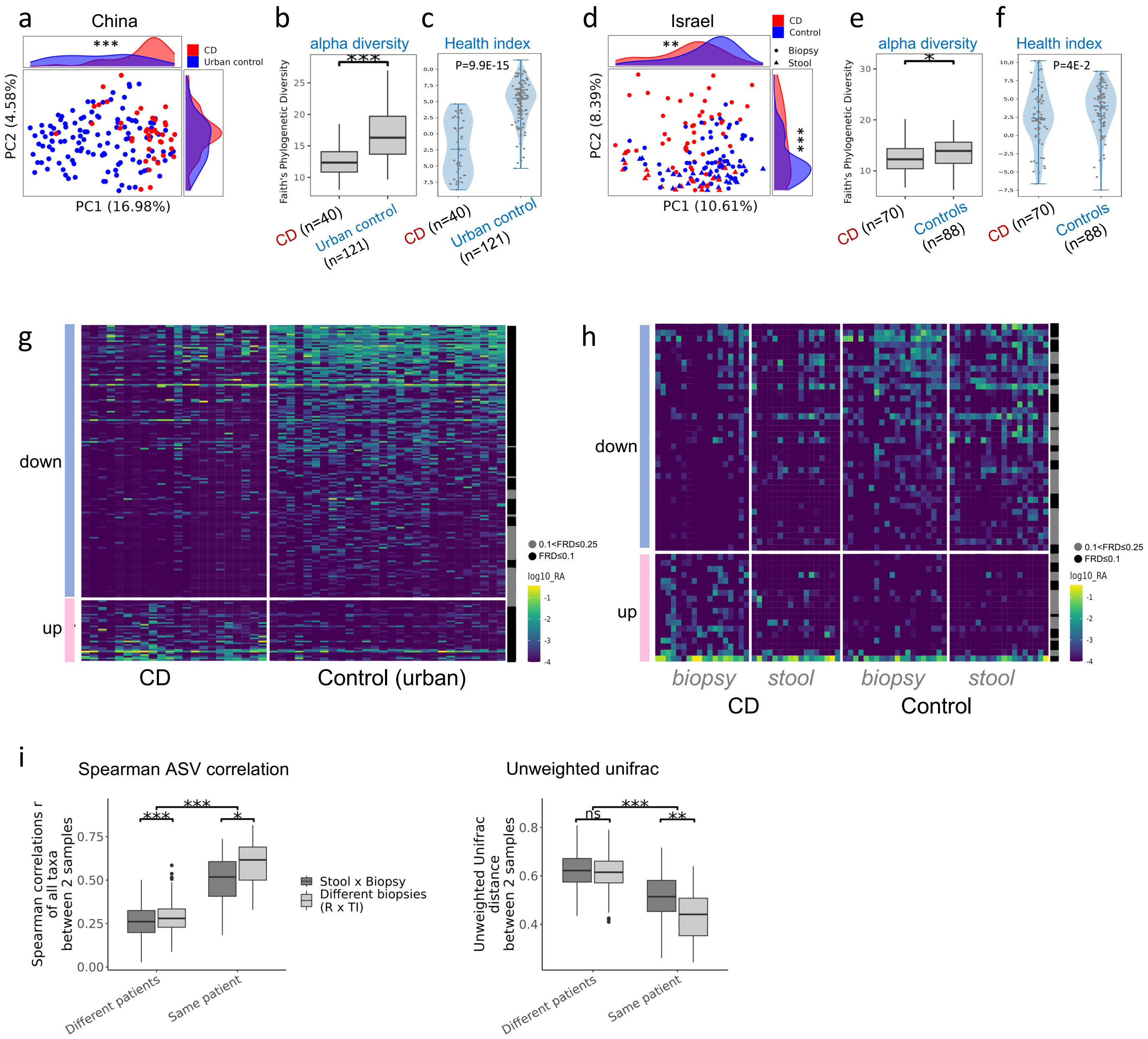

Supplementary Fig. 4. CD-increased taxa are enriched in mucosal biopsies, with overall persistence along the ileum, rectum, and stool within subjects. a. Unweighted UniFrac PCoA plot of 161 urban China 16S stool samples, colored by diagnosis. Histograms show the distribution of samples and diagnosis on PC1 and PC2. **b.** Boxplots of alpha diversity values, using Faith's phylogenetic diversity, between CD (n=40) and urban controls (n=121, Mann-Whitney p. value = 2.56e-8). **c.** Violin plot of our previously defined health index between CD (n=40) and urban (n=121, Mann-Whitney p. value = 5.28e-15). **d.** Unweighted UniFrac PCoA plot of 158 Israel 16S stool and biopsy samples, with color representing diagnosis and shape separating stool and biopsy. Histograms show the distribution of samples and diagnosis on PC1 and PC2. **e.** Boxplots of alpha diversity values of Israel 16S stool and biopsy samples, using Faith's phylogenetic diversity, between CD (n=70) and control (n=88, Mann-Whitney p. value = 0.04). **f.** Violin plot of our previously defined health index between CD (n=70) and controls (n=88). **g.** A heatmap showing ASVs significantly different between China CD and urban controls ($p<0.05$, FDR $\leq$ 0.25), using a maaslin2 analysis controlling for age and gender. Each row represents a different ASV, ordered by

the effect size, and each column a different sample (list in **Supplementary Dataset5**). **h.** A heatmap showing ASVs significantly different between Israel CD and controls (FDR $\leq$ 0.25, black mark on the right indicates those with FDR$\leq$0.1), using a maaslin2 analysis controlling for age, gender, sample type (stool or biopsy), and patient ID as the random variable. **i.** Boxplots of the distances between stool vs. biopsy samples, and different biopsies (rectum vs. terminal ileum), and between the same patient (stool vs. biopsy n=42, different biopsies n=27) and different patient's samples (stool vs. biopsy n=958, different biopsies n=598). Two complementing measures were used for the distance calculations: Spearman's rho for the correlations of all ASV's relative abundance between every two samples (left), and unweighted unifrac distance (right). ns=not significant, *p< 0.05, **p<0.01, ***p<0.001. Box plot center line and limit; median, upper and lower quartiles; whiskers, 1.5x interquartile range.

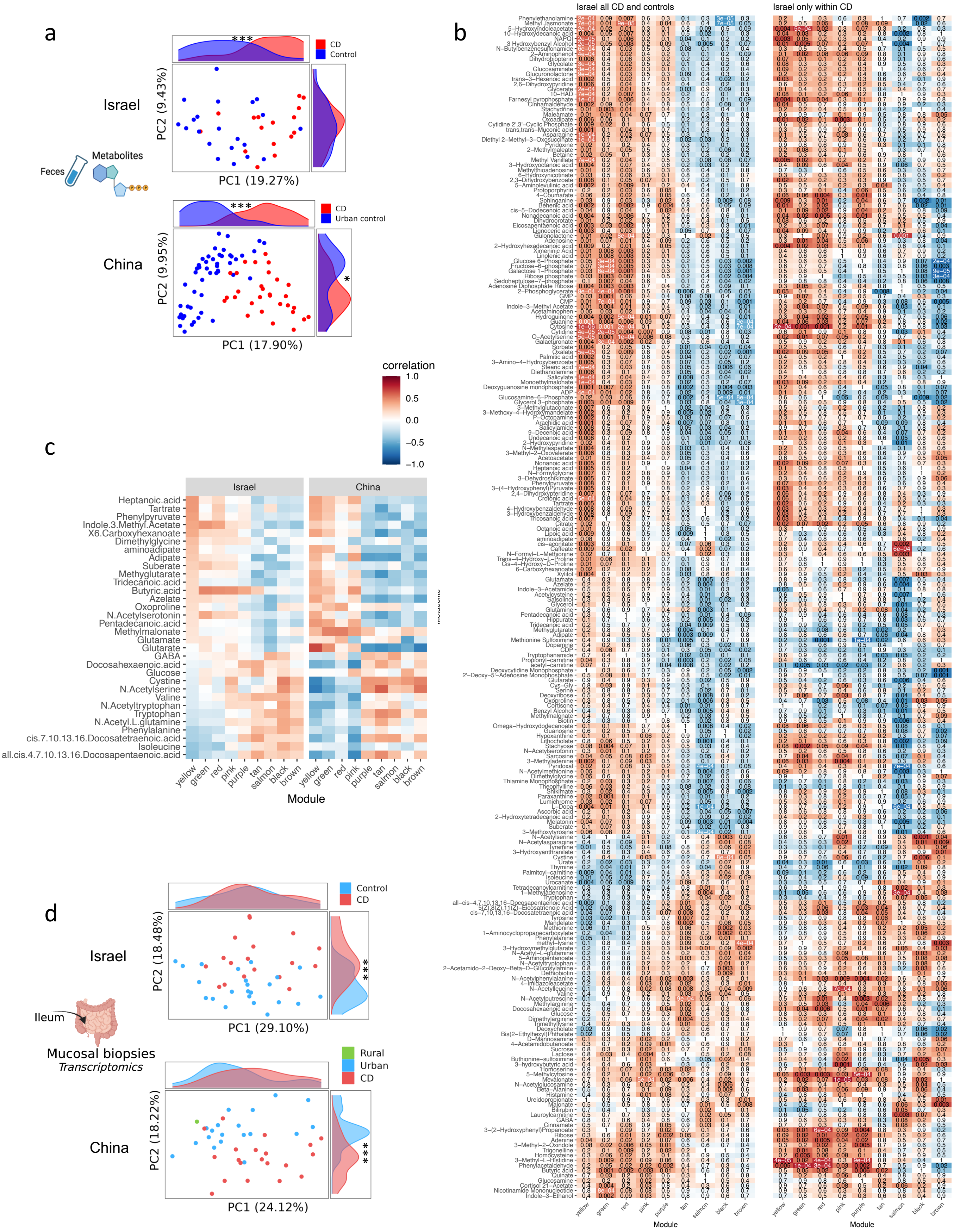

Supplementary Fig. 5. Correlations with fecal metabolomics. a. Canberra distance PCoA plot of Israel (up) and China (down) stool metabolites samples, colored by diagnosis. Histograms show the distribution of samples and diagnosis on PC1 and PC2.

b. Detailed heatmaps of the correlation between each ileal Israeli transcriptomics module and stool metabolites, Colored by correlation coefficient. left – using all samples (n=41), right – using only CD samples (n=18, 97.5% of correlations showed the same direction within CD as in the overall cohort, with binomial test $p < 2.2e-16$). Only metabolites with Benjamin-Hochberg FDR ≤ 0.25 in at least one module calculated on all the samples are shown. **c**. detailed heatmap of Fig. 5F. Heatmap of the correlations between each module and the 32 stool metabolites (of 91 common metabolites in the SYS and Sheba datasets) that showed significant correlation in the same direction with CD in Israel and in China, colored by correlation coefficient. A metabolites direction here is defined as the direction of the strongest correlation between all the modules. **d.** PCA of ileal transcriptomics genes in China and Israel. *$p< 0.05$, **$p<0.01$, ***$p<0.001$, Mann-Whitney test.

a. Black

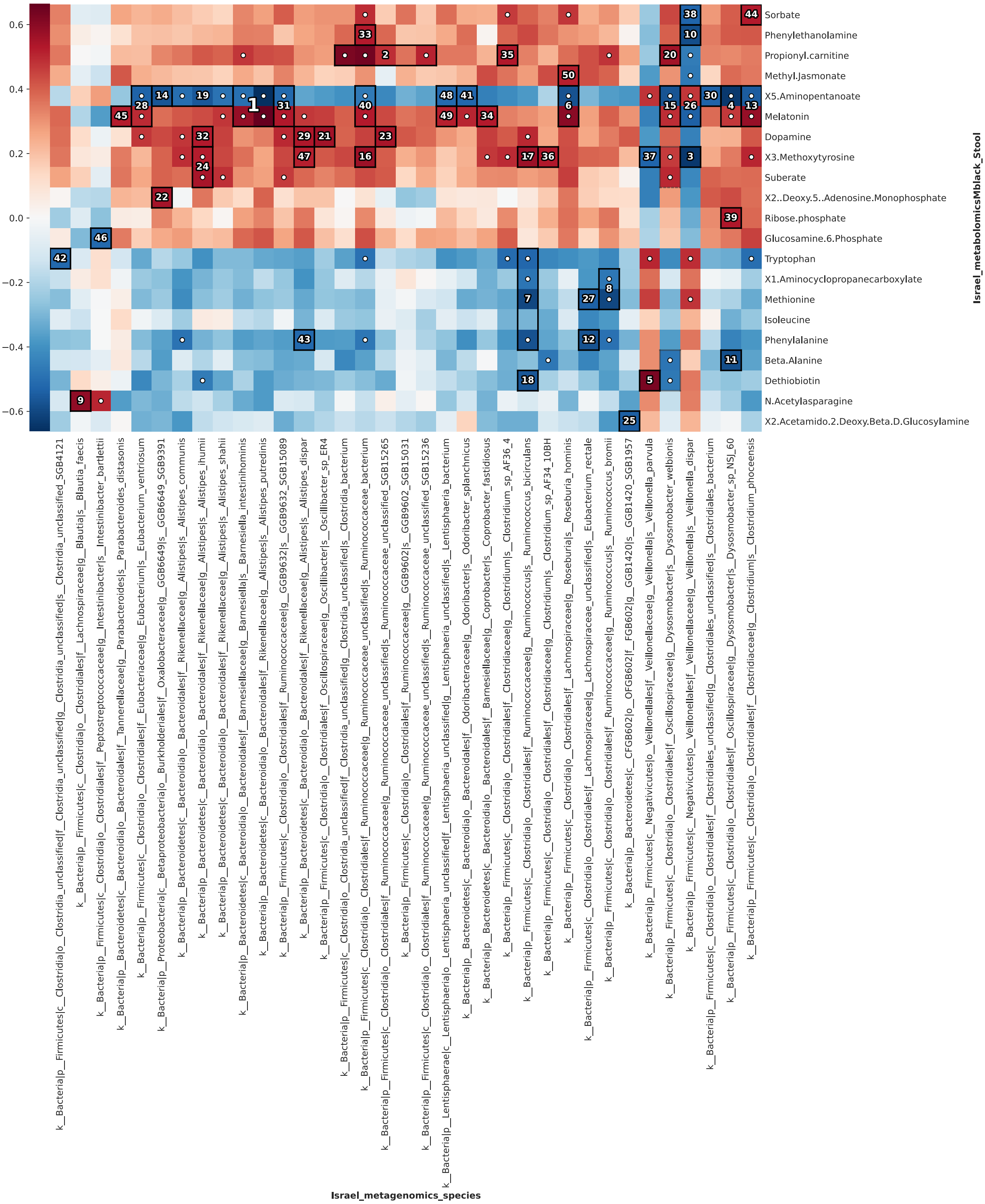
0.6
0.4
0.2
0.0
−0.2
−0.4
−0.6
Sorbate
Phenylethanolamine
Propionyl.carnitine
Methyl.Jasmonate
X5.Aminopentanoate
Melatonin
Dopamine
X3.Methoxytyrosine
Suberate
X2..Deoxy.5..Adenosine.Monophosphate
Ribose.phosphate
Glucosamine.6.Phosphate
Tryptophan
X1.Aminocyclopropanecarboxylate
Methionine
Isoleucine
Phenylalanine
Beta.Alanine
Dethiobiotin
N.Acetylasparagine
X2.Acetamido.2.Deoxy.Beta.D.Glucosylamine
Israel_metabolomicsMblack_Stool
k__Bacteria|p__Firmicutes|c__Clostridia|o__Clostridia_unclassified|f__Clostridia_unclassified|g__Clostridia_unclassified|s__Clostridia_unclassified_SGB4121
k__Bacteria|p__Firmicutes|c__Clostridia|o__Clostridiales|f__Lachnospiraceae|g__Blautia|s__Blautia_faecis
k__Bacteria|p__Firmicutes|c__Clostridia|o__Clostridiales|f__Peptostreptococcaceae|g__Intestinibacter|s__Intestinibacter_bartlettii
k__Bacteria|p__Bacteroidetes|c__Bacteroidia|o__Bacteroidales|f__Tannerellaceae|g__Parabacteroides|s__Parabacteroides_distasonis
k__Bacteria|p__Firmicutes|c__Clostridia|o__Clostridiales|f__Eubacteriaceae|g__Eubacterium|s__Eubacterium_ventriosum
k__Bacteria|p__Proteobacteria|c__Betaproteobacteria|o__Burkholderiales|f__Oxalobacteraceae|g__GGB6649|s__GGB6649_SGB9391
k__Bacteria|p__Bacteroidetes|c__Bacteroidia|o__Bacteroidales|f__Rikenellaceae|g__Alistipes|s__Alistipes_communis
k__Bacteria|p__Bacteroidetes|c__Bacteroidia|o__Bacteroidales|f__Rikenellaceae|g__Alistipes|s__Alistipes_ihumii
k__Bacteria|p__Bacteroidetes|c__Bacteroidia|o__Bacteroidales|f__Rikenellaceae|g__Alistipes|s__Alistipes_shahii
k__Bacteria|p__Bacteroidetes|c__Bacteroidia|o__Bacteroidales|f__Barnesiellaceae|g__Barnesiella|s__Barnesiella_intestinihominis
k__Bacteria|p__Bacteroidetes|c__Bacteroidia|o__Bacteroidales|f__Rikenellaceae|g__Alistipes|s__Alistipes_putredinis
k__Bacteria|p__Firmicutes|c__Clostridia|o__Clostridiales|f__Ruminococcaceae|g__GGB9632|s__GGB9632_SGB15089
k__Bacteria|p__Bacteroidetes|c__Bacteroidia|o__Bacteroidales|f__Rikenellaceae|g__Alistipes|s__Alistipes_dispar
k__Bacteria|p__Firmicutes|c__Clostridia|o__Clostridiales|f__Oscillospiraceae|g__Oscillibacter|s__Oscillibacter_sp_ER4
k__Bacteria|p__Firmicutes|c__Clostridia|o__Clostridia_unclassified|f__Clostridia_unclassified|g__Clostridia_unclassified|s__Clostridia_bacterium
k__Bacteria|p__Firmicutes|c__Clostridia|o__Clostridiales|f__Ruminococcaceae|g__Ruminococcaceae_unclassified|s__Ruminococcaceae_bacterium
k__Bacteria|p__Firmicutes|c__Clostridia|o__Clostridiales|f__Ruminococcaceae|g__Ruminococcaceae_unclassified|s__Ruminococcaceae_unclassified_SGB15265
k__Bacteria|p__Firmicutes|c__Clostridia|o__Clostridiales|f__Ruminococcaceae|g__GGB9602|s__GGB9602_SGB15031
k__Bacteria|p__Firmicutes|c__Clostridia|o__Clostridiales|f__Ruminococcaceae|g__Ruminococcaceae_unclassified|s__Ruminococcaceae_unclassified_SGB15236
k__Bacteria|p__Lentisphaerae|c__Lentisphaeria|o__Lentisphaeria_unclassified|f__Lentisphaeria_unclassified|g__Lentisphaeria_unclassified|s__Lentisphaeria_bacterium
k__Bacteria|p__Bacteroidetes|c__Bacteroidia|o__Bacteroidales|f__Odoribacteraceae|g__Odoribacter|s__Odoribacter_splanchnicus
k__Bacteria|p__Bacteroidetes|c__Bacteroidia|o__Bacteroidales|f__Barnesiellaceae|g__Coprobacter|s__Coprobacter_fastidiosus
k__Bacteria|p__Firmicutes|c__Clostridia|o__Clostridiales|f__Clostridiaceae|g__Clostridium|s__Clostridium_sp_AF36_4
k__Bacteria|p__Firmicutes|c__Clostridia|o__Clostridiales|f__Ruminococcaceae|g__Ruminococcus|s__Ruminococcus_bicirculans
k__Bacteria|p__Firmicutes|c__Clostridia|o__Clostridiales|f__Clostridiaceae|g__Clostridium|s__Clostridium_sp_AF34_10BH
k__Bacteria|p__Firmicutes|c__Clostridia|o__Clostridiales|f__Lachnospiraceae|g__Roseburia|s__Roseburia_hominis
k__Bacteria|p__Firmicutes|c__Clostridia|o__Clostridiales|f__Lachnospiraceae|g__Lachnospiraceae_unclassified|s__Eubacterium_rectale
k__Bacteria|p__Firmicutes|c__Clostridia|o__Clostridiales|f__Ruminococcaceae|g__Ruminococcus|s__Ruminococcus_bromii
k__Bacteria|p__Bacteroidetes|c__CFGB602|o__OFGB602|f__FGB602|g__GGB1420|s__GGB1420_SGB1957
k__Bacteria|p__Firmicutes|c__Negativicutes|o__Veillonellales|f__Veillonellaceae|g__Veillonella|s__Veillonella_parvula
k__Bacteria|p__Firmicutes|c__Clostridia|o__Clostridiales|f__Oscillospiraceae|g__Dysosmobacter|s__Dysosmobacter_welbionis
k__Bacteria|p__Firmicutes|c__Negativicutes|o__Veillonellales|f__Veillonellaceae|g__Veillonella|s__Veillonella_dispar
k__Bacteria|p__Firmicutes|c__Clostridia|o__Clostridiales|f__Clostridiales_unclassified|g__Clostridiales_unclassified|s__Clostridiales_bacterium
k__Bacteria|p__Firmicutes|c__Clostridia|o__Clostridiales|f__Oscillospiraceae|g__Dysosmobacter|s__Dysosmobacter_sp_NSJ_60
k__Bacteria|p__Firmicutes|c__Clostridia|o__Clostridiales|f__Clostridiaceae|g__Clostridium|s__Clostridium_phoceensis
Israel_metagenomics_species

b. Brown

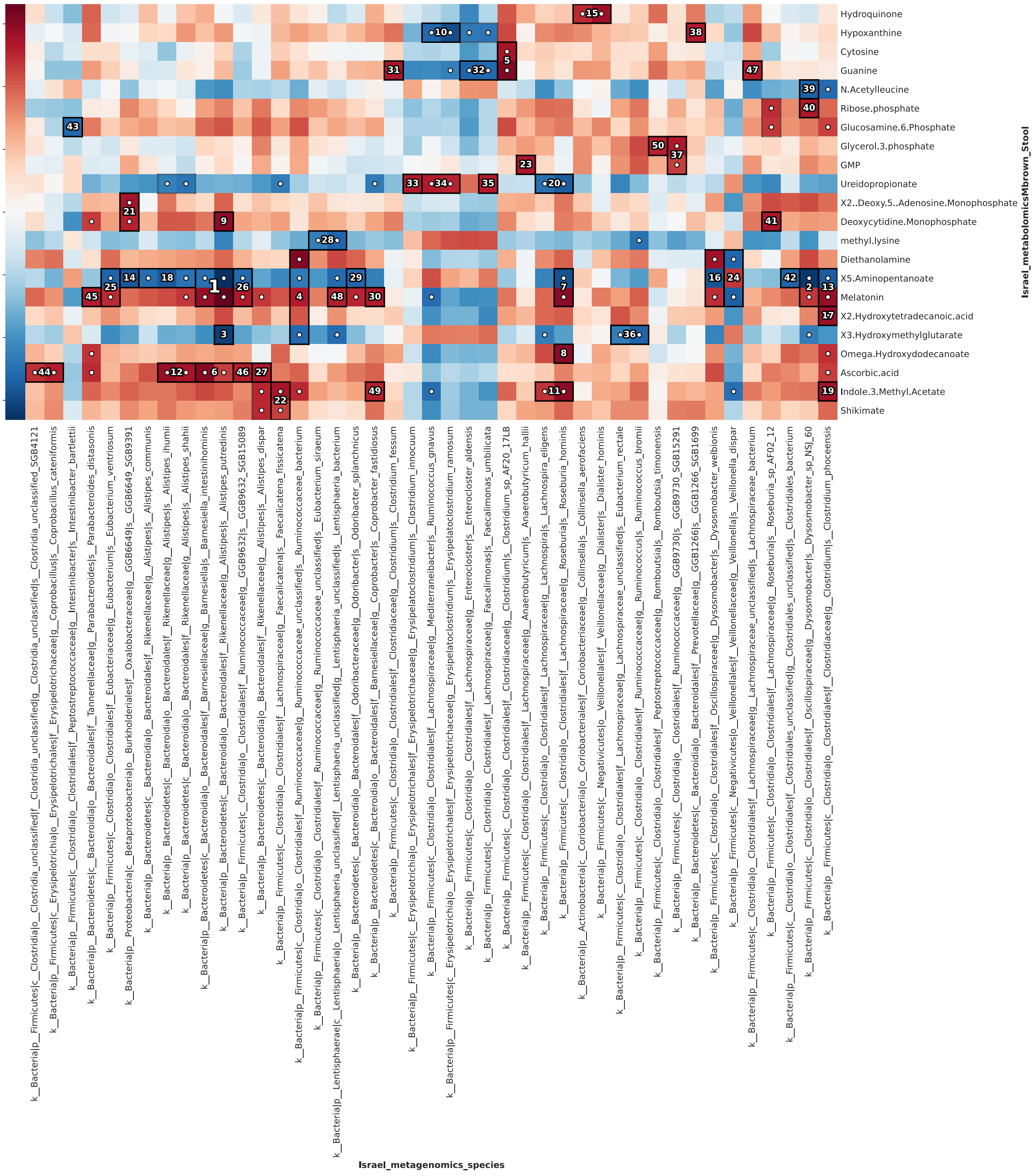
0.6
0.4
0.2
0.0
−0.2
−0.4
−0.6
Hydroquinone
Hypoxanthine
Cytosine
Guanine
N.Acetylleucine
Ribose.phosphate
Glucosamine.6.Phosphate
Glycerol.3.phosphate
GMP
Ureidopropionate
X2..Deoxy.5..Adenosine.Monophosphate
Deoxycytidine.Monophosphate
methyl.lysine
Diethanolamine
X5.Aminopentanoate
Melatonin
X2.Hydroxytetradecanoic.acid
X3.Hydroxymethylglutarate
Omega.Hydroxydodecanoate
Ascorbic.acid
Indole.3.Methyl.Acetate
Shikimate
Israel_metabolomicsMbrown_Stool
k__Bacteria|p__Firmicutes|c__Clostridia|o__Clostridia_unclassified|f__Clostridia_unclassified|g__Clostridia_unclassified|s__Clostridia_unclassified_SGB4121
k__Bacteria|p__Firmicutes|c__Erysipelotrichia|o__Erysipelotrichales|f__Erysipelotrichaceae|g__Coprobacillus|s__Coprobacillus_cateniformis
k__Bacteria|p__Firmicutes|c__Clostridia|o__Clostridiales|f__Peptostreptococcaceae|g__Intestinibacter|s__Intestinibacter_bartlettii
k__Bacteria|p__Bacteroidetes|c__Bacteroidia|o__Bacteroidales|f__Tannerellaceae|g__Parabacteroides|s__Parabacteroides_distasonis
k__Bacteria|p__Firmicutes|c__Clostridia|o__Clostridiales|f__Eubacteriaceae|g__Eubacterium|s__Eubacterium_ventriosum
k__Bacteria|p__Proteobacteria|c__Betaproteobacteria|o__Burkholderiales|f__Oxalobacteraceae|g__GGB6649|s__GGB6649_SGB9391
k__Bacteria|p__Bacteroidetes|c__Bacteroidia|o__Bacteroidales|f__Rikenellaceae|g__Alistipes|s__Alistipes_communis
k__Bacteria|p__Bacteroidetes|c__Bacteroidia|o__Bacteroidales|f__Rikenellaceae|g__Alistipes|s__Alistipes_ihumii
k__Bacteria|p__Bacteroidetes|c__Bacteroidia|o__Bacteroidales|f__Rikenellaceae|g__Alistipes|s__Alistipes_shahii
k__Bacteria|p__Bacteroidetes|c__Bacteroidia|o__Bacteroidales|f__Barnesiellaceae|g__Barnesiella|s__Barnesiella_intestinihominis
k__Bacteria|p__Bacteroidetes|c__Bacteroidia|o__Bacteroidales|f__Rikenellaceae|g__Alistipes|s__Alistipes_putredinis
k__Bacteria|p__Firmicutes|c__Clostridia|o__Clostridiales|f__Ruminococcaceae|g__GGB9632|s__GGB9632_SGB15089
k__Bacteria|p__Bacteroidetes|c__Bacteroidia|o__Bacteroidales|f__Rikenellaceae|g__Alistipes|s__Alistipes_dispar
k__Bacteria|p__Firmicutes|c__Clostridia|o__Clostridiales|f__Lachnospiraceae|g__Faecalicatena|s__Faecalicatena_fissicatena
k__Bacteria|p__Firmicutes|c__Clostridia|o__Clostridiales|f__Ruminococcaceae|g__Ruminococcaceae_unclassified|s__Ruminococcaceae_bacterium
k__Bacteria|p__Firmicutes|c__Clostridia|o__Clostridiales|f__Ruminococcaceae|g__Eubacterium|s__Eubacterium_siraeum
k__Bacteria|p__Lentisphaerae|c__Lentisphaeria|o__Lentisphaeria_unclassified|f__Lentisphaeria_unclassified|g__Lentisphaeria_unclassified|s__Lentisphaeria_bacterium
k__Bacteria|p__Bacteroidetes|c__Bacteroidia|o__Bacteroidales|f__Odoribacteraceae|g__Odoribacter|s__Odoribacter_splanchnicus
k__Bacteria|p__Bacteroidetes|c__Bacteroidia|o__Bacteroidales|f__Barnesiellaceae|g__Coprobacter|s__Coprobacter_fastidiosus
k__Bacteria|p__Firmicutes|c__Clostridia|o__Clostridiales|f__Clostridiaceae|g__Clostridium|s__Clostridium_fessum
k__Bacteria|p__Firmicutes|c__Erysipelotrichia|o__Erysipelotrichales|f__Erysipelotrichaceae|g__Erysipelatoclostridium|s__Clostridium_innocuum
k__Bacteria|p__Firmicutes|c__Clostridia|o__Clostridiales|f__Lachnospiraceae|g__Mediterraneibacter|s__Ruminococcus_gnavus
k__Bacteria|p__Firmicutes|c__Erysipelotrichia|o__Erysipelotrichales|f__Erysipelotrichaceae|g__Erysipelatoclostridium|s__Erysipelatoclostridium_ramosum
k__Bacteria|p__Firmicutes|c__Clostridia|o__Clostridiales|f__Lachnospiraceae|g__Enterocloster|s__Enterocloster_aldensis
k__Bacteria|p__Firmicutes|c__Clostridia|o__Clostridiales|f__Lachnospiraceae|g__Faecalimonas|s__Faecalimonas_umbilicata
k__Bacteria|p__Firmicutes|c__Clostridia|o__Clostridiales|f__Clostridiaceae|g__Clostridium|s__Clostridium_sp_AF20_17LB
k__Bacteria|p__Firmicutes|c__Clostridia|o__Clostridiales|f__Lachnospiraceae|g__Anaerobutyricum|s__Anaerobutyricum_hallii
k__Bacteria|p__Firmicutes|c__Clostridia|o__Clostridiales|f__Lachnospiraceae|g__Lachnospira|s__Lachnospira_eligens
k__Bacteria|p__Firmicutes|c__Clostridia|o__Clostridiales|f__Lachnospiraceae|g__Roseburia|s__Roseburia_hominis
k__Bacteria|p__Actinobacteria|c__Coriobacteriia|o__Coriobacteriales|f__Coriobacteriaceae|g__Collinsella|s__Collinsella_aerofaciens
k__Bacteria|p__Firmicutes|c__Negativicutes|o__Veillonellales|f__Veillonellaceae|g__Dialister|s__Dialister_hominis
k__Bacteria|p__Firmicutes|c__Clostridia|o__Clostridiales|f__Lachnospiraceae|g__Lachnospiraceae_unclassified|s__Eubacterium_rectale
k__Bacteria|p__Firmicutes|c__Clostridia|o__Clostridiales|f__Ruminococcaceae|g__Ruminococcus|s__Ruminococcus_bromii
k__Bacteria|p__Firmicutes|c__Clostridia|o__Clostridiales|f__Peptostreptococcaceae|g__Romboutsia|s__Romboutsia_timonensis
k__Bacteria|p__Firmicutes|c__Clostridia|o__Clostridiales|f__Ruminococcaceae|g__GGB9730|s__GGB9730_SGB15291
k__Bacteria|p__Bacteroidetes|c__Bacteroidia|o__Bacteroidales|f__Prevotellaceae|g__GGB1266|s__GGB1266_SGB1699
k__Bacteria|p__Firmicutes|c__Clostridia|o__Clostridiales|f__Oscillospiraceae|g__Dysosmobacter|s__Dysosmobacter_welbionis
k__Bacteria|p__Firmicutes|c__Negativicutes|o__Veillonellales|f__Veillonellaceae|g__Veillonella|s__Veillonella_dispar
k__Bacteria|p__Firmicutes|c__Clostridia|o__Clostridiales|f__Lachnospiraceae|g__Lachnospiraceae_unclassified|s__Lachnospiraceae_bacterium
k__Bacteria|p__Firmicutes|c__Clostridia|o__Clostridiales|f__Lachnospiraceae|g__Roseburia|s__Roseburia_sp_AF02_12
k__Bacteria|p__Firmicutes|c__Clostridia|o__Clostridiales_unclassified|f__Clostridiales_unclassified|g__Clostridiales_unclassified|s__Clostridiales_bacterium
k__Bacteria|p__Firmicutes|c__Clostridia|o__Clostridiales|f__Oscillospiraceae|g__Dysosmobacter|s__Dysosmobacter_sp_NSJ_60
k__Bacteria|p__Firmicutes|c__Clostridia|o__Clostridiales|f__Clostridiaceae|g__Clostridium|s__Clostridium_phoceensis
Israel_metagenomics_species

c. Salmon

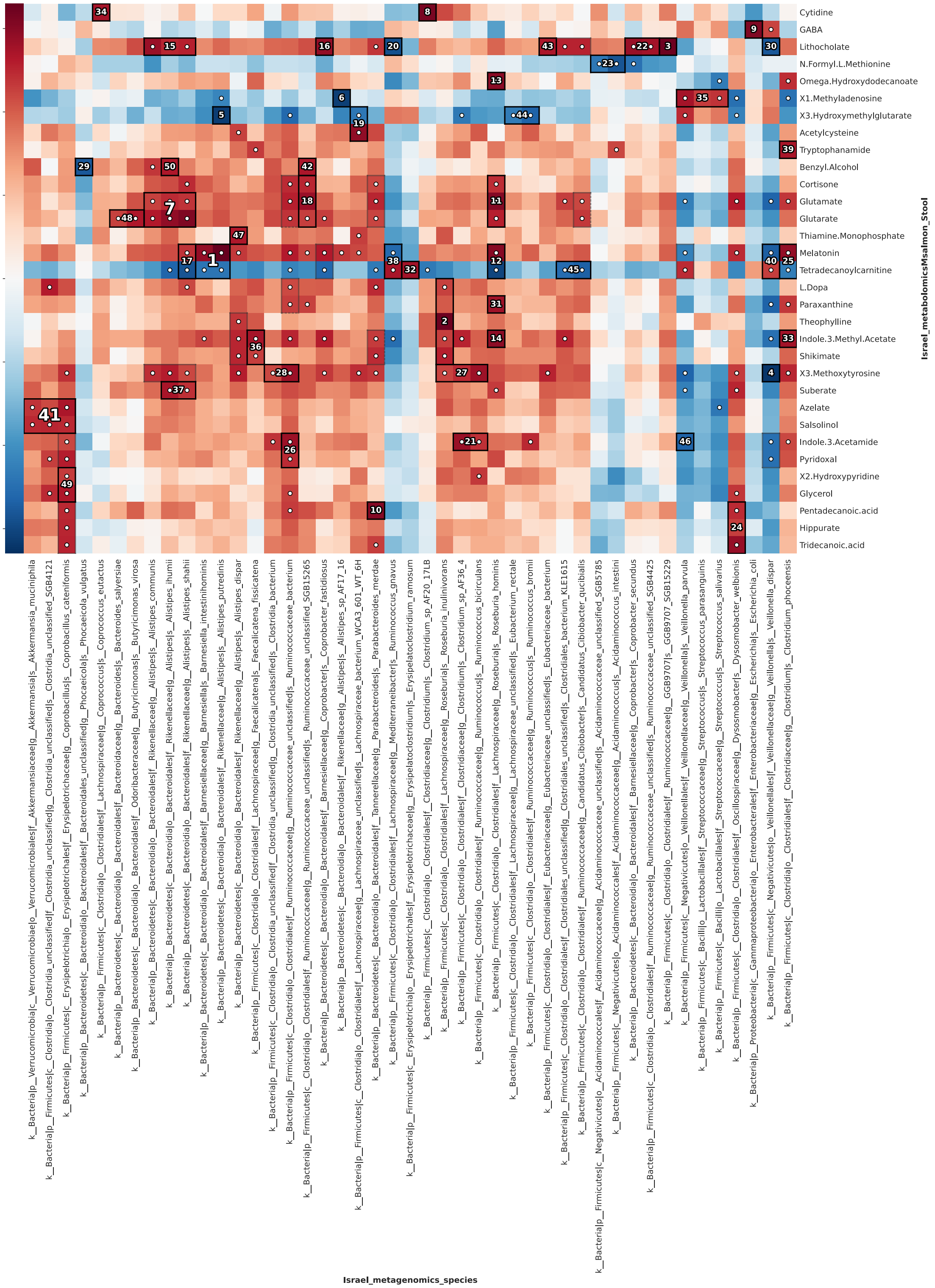

Supplementary Fig. 6. Microbial shotgun taxa correlations with fecal metabolomics. Heatmap of HAllA correlations between fecal metabolites linked with black (**a**), brown (**b**), and salmon (**c**) immune modules, and metagenomics taxa. Color represents Spearman's rho and significant correlations (FDR ≤ 0.25) are marked.

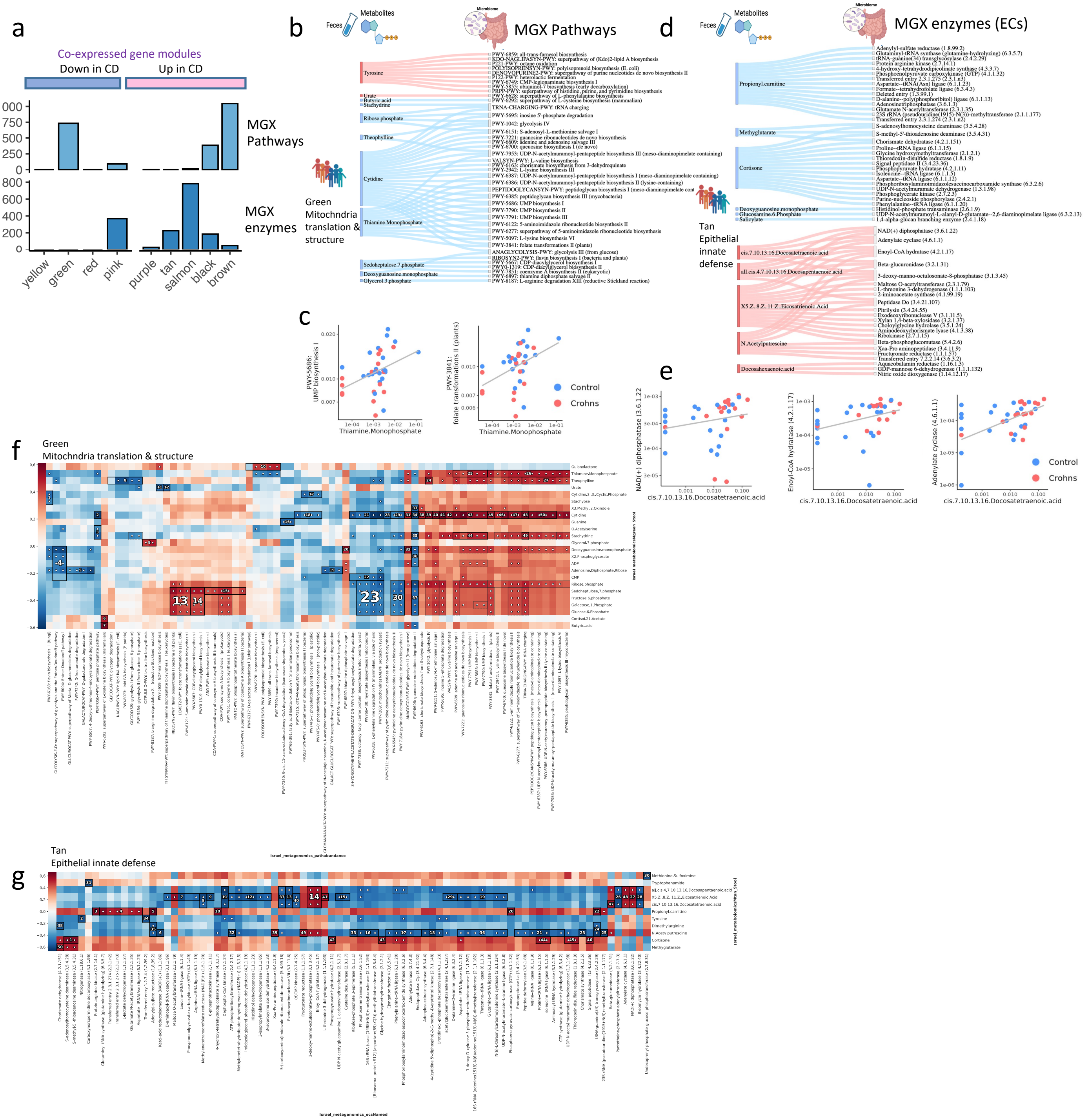

Supplementary Fig. 7. **Examples of fecal metabolites associations with pathways and ECs**. The correlations between metabolites associated with each ileal transcriptomics module in the Israeli subcohort were tested against microbiome data separately using HAllA (Hierarchical All-against-All Association Testing) with FDR ≤ 0.25. **a.** Bar plot of the number of significant correlations between metabolites associated with the different modules and MGX pathways and MGX enzymes (ECs)**.** Sankey figure of up to top 50 significant correlations for control-associated metabolites (blue) and disease-associated metabolites (red) for correlations between green module-associated metabolites and MGX pathways **(b)**, and tan module-associated metabolites and MGX ECs **(d)** with example scatterplots for metabolites and **(c)** MGX pathways, **(e)** MGX ECs. **f-g**. Heatmap of HAllA correlations between fecal metabolites linked with green (**f**), and tan (**g**), and metagenomics pathways and EC respectively. Color represents Spearman's rho and significant correlations (FDR ≤ 0.25) are marked.
