# Supplementary material for: Diet-omics in the Study of Urban and Rural Crohn disease Evolution (SOURCE) cohort
Source: Nat Commun. 2024 May 4;15:3764. doi: 10.1038/s41467-024-48106-6 (PMC11069498; doi:10.1038/s41467-024-48106-6)
Supplement: Supplementary file 3 — Description of Additional Supplementary Files [file 41467_2024_48106_MOESM3_ESM.pdf]

**Title:** Supplementary Dataset 1:
**Description:** SOURCE cohort metadata and data availability, detailed FFQ and questionnaire tables, and a STORMS checklist. Datasheet, including data for Fig. 1 and Supplementary Fig 2.

**Title:** Supplementary Dataset 2:
**Description:** 16S taxonomy China and Israel datasets. Datasheet, including data for Fig. 2.

**Title:** Supplementary Dataset 3:
**Description:** Rural and rural-urban associated 16S bacteria and metabolites, and within all rural correlations between metabolites, 16S bacteria, and FFQ components. Datasheet, including data for Fig. 2 and Supplementary Fig. 1.

**Title:** Supplementary Dataset 4:
**Description:** 16S microbial variance explained by FFQ and questionnaire data, and specific bacteria associated with China control fat and iron consumption. Datasheet, including data for Fig. 3 and Supplementary Fig. 3.

**Title:** Supplementary Dataset 5:
**Description:** 16S bacteria significantly different between CD and control in Israel and China. Datasheet, including data used in Supplementary Fig. 4.

**Title:** Supplementary Dataset 6:
**Description:** Ileal transcriptomics WGCNA modules genes, functional annotation enrichment results, and correlations to metadata, FFQ. Datasheet, including data used in Fig. 4 and Supplementary Fig. 5.

**Title:** Supplementary Dataset 7:
**Description:** Fecal metabolomics China and Israel datasets, and a list of common metabolites between the two. Datasheet, including data used in Fig. 4.

**Title:** Supplementary Dataset 8:
**Description:** Metabolites, as well as module-associated metabolites correlations with FFQ, 16S bacteria, and MGX (Fig. 5). sPLS Spearman correlation p-values and rhos (Fig. 5e-f) and the 67 features across omics that were associated with CD (Fig. 5g). Datasheet, including data for Fig. 5 and Supplementary Fig. 6&7.

**Title:** Supplementary Dataset 9:
**Description:** Integration of the different omics while accounting for the shared variation and their association with CD. An interactive version of this plot is in Fig. 5h.
