# Supplementary material for: Diet-omics in the Study of Urban and Rural Crohn disease Evolution (SOURCE) cohort
Source: Nat Commun. 2024 May 4;15:3764. doi: 10.1038/s41467-024-48106-6 (PMC11069498; doi:10.1038/s41467-024-48106-6)
Supplement: Supplementary file 12 — Supplementary Dataset 9 [file 41467_2024_48106_MOESM12_ESM.html]

plotly
¶
¶
¶
¶
¶
¶
¶
